# Stress of conscience in healthcare in turbulent times: A longitudinal study

**DOI:** 10.1177/09697330231204949

**Published:** 2023-10-27

**Authors:** Mikko Taipale, Mari Herttalampi, Joona Muotka, Saija Mauno, Taru Feldt

**Affiliations:** 4168 University of Jyväskylä, Finland; 4168 University of Jyväskylä, Finland; Tampere University, Finland; 4168 University of Jyväskylä, Finland

**Keywords:** Stress of conscience, long-term work intensification, change management, COVID-19, healthcare

## Abstract

**Background:**

Healthcare workers frequently face ethically demanding situations in their work, potentially leading to stress of conscience. Long-term work intensification (more and more effort demanded year after year), organizational change and COVID-19 may be risk factors concerning stress of conscience.

**Aims:**

The main aim was to investigate the relationship between long-term work intensification and stress of conscience among the personnel in a healthcare organization. Organizational change management was considered a mediator and COVID-19-related work stress a moderator in the association between work intensification and stress of conscience.

**Research design, participants and context:**

A total of 211 healthcare district employees participated in a longitudinal survey using questionnaires collected in 2019 (major organizational change in the planning stage) and 2021 (organizational change completed).

**Ethical considerations:**

The study was implemented according to the guidelines of the Finnish National Board on Research Integrity. The Finnish instructions were that no review by an ethics committee was necessary because participation was voluntary, informed consent was requested, participants were assured that they were free to withdraw from the longitudinal study at any time and no health data were collected.

**Findings:**

Long-term work intensification was associated with more severe stress of conscience. Long-term work intensification was partially mediated through change management to stress of conscience. High COVID-19 stress strengthened the association between long-term work intensification and stress of conscience.

**Conclusions:**

Long-term work intensification must be addressed to reduce stress of conscience in healthcare, otherwise the healthcare system will be vulnerable to changes and crisis. Extra resources for personnel and management should be allocated because of work intensification during organizational change and health crises like the COVID-19 pandemic to alleviate stress of conscience.

## Introduction

Healthcare workers’ aim primarily at what is morally good or right for patients.^1^ However, in turbulent working environments with increasing demands, changes and crises ‘doing good’ and upholding ethical principles is challenging.^2^ The acceleration of working life due to rapid technological development and digitalization has led to an intensification of work in healthcare: increased workload, time pressures, pace of work and multitasking demands.^3-6^ Simultaneously, many healthcare organizations are endeavouring to become more productive and ensure their sustainability and resource sufficiency.^7,8^ In organizational transition, change management must be able to ensure employees adequate resources to secure their commitment to the change.^9^ Finally, the global COVID-19 pandemic has tested health sector resources and its workers’ performance unprecedentedly.^2,10^ Such cross-pressure from multiple sources tests workers’ ability and resources to uphold ethical principles and standards, which may lead, in turn, to high stress of conscience.^11^

This two-year longitudinal study conducted in a large healthcare organization undergoing major organizational change during follow-up investigated how work intensification, organizational change management and COVID-19-related work stress pandemic are associated with stress of conscience among healthcare personnel. At the beginning of the study, in 2019, this organizational change was being planned and at the second measurement point, 2 years later, in 2021, the change had been completed (a new hospital building, a new patient-centred service and care processes were in place). Between the data collection points, the COVID-19 pandemic spread, impacting the healthcare sector broadly also in Finland.^12^

**Figure 1.**
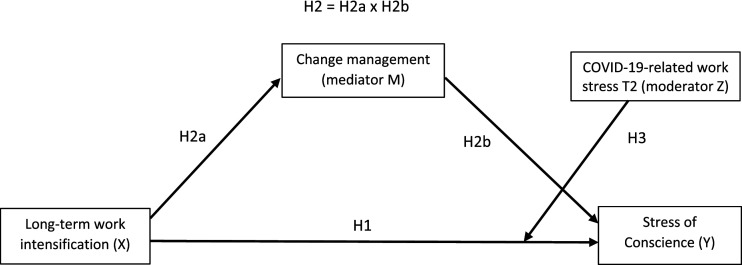
Conceptual model of the relationships between long-term work intensification, change management, COVID-19-related work stress and stress of conscience among healthcare personnel.

The results of this study can be used to find ways to manage healthcare workers' stress of conscience in an ever-intensifying working life fraught with organizational changes and sudden crises. In addition, the results regarding stress of conscience may be used as indicators of system improvements and interventions to promote ethically and morally sustainable patient care. The conceptual model of the study is presented in Figure 1. Next, we present our detailed hypotheses based on theory and existing research.

### Stress of conscience as a result of long-term work intensification

*Stress of conscience* is defined ‘a product of the frequency of the stressful situation and of the perceived degree of troubled conscience as rated by healthcare workers themselves’.^1(p636)^ Consequently, stress of conscience arises in situations in which workers in patient care cannot act in accordance with their consciences and are unable to process or resolve the ethical dilemma involved.^1^ Such situations may include tasks where workers must lower their aspirations to provide good care or when time does not suffice to provide the care a patient needs.^1^ Stress of conscience therefore has to do with healthcare workers’ realization that they cannot come up to their own ethical standards while working with patients.^11^

Stress of conscience as a concept is close moral dilemmas and moral stress, but it has its own emphasis. Moral dilemma refers to a situation in which there is difficulty in choosing between two or more moral values when caring for a patient.^13^ Instead of a choice between moral values, stress of conscience refers to the frequency and magnitude of a troubled conscience arising from ethically challenging situations.^11^ Moral distress refers to suffering experienced by healthcare workers as a result of restricted moral agency.^14^ It typically develops in situations where ‘one knows the right thing to do, but institutional constraints make it nearly impossible to pursue the right course of action’ according to Jameton’s research cited in Mänttäri.^14^ Compared to moral distress, stress of conscience includes a wider range of situations in patient care and the magnitude of a troubled conscience.^11^

The theoretical roots of stress of conscience are in the conceptualization of conscience as an inner voice, the concept of moral sense and transactional stress theory defining psychological stress as result of interaction between the individual and the environment.^1^ Therefore, stress of conscience appears when the ethical demands of the situation or the moral expectations of the environment are perceived to exceed the individual’s personal resources and their moral sense activates the inner voice noting an offence against the individual’s moral goals.^1^ In healthcare, the demands are the moral goals of working with patients – especially providing good care and protecting patients from harm.^11^When these moral goals cannot be achieved, the personnel experiences stress of conscience.^11^ When healthcare workers feel compelled to compromise on good care or deliver low quality care, that is, due to shortage of time, their stress of conscience increases.^11^

*Work intensification* is a job stressor attributable to the widespread acceleration in the pace of working life.^4,6,15^ Typically, work intensification refers to increases in quantitative workload (e.g. in terms of increased time pressures, pace of work and multitasking demands) requiring more and more effort from employees to accomplish their tasks.^3,5,6^ Therefore, work intensification may prevent the achievement of the moral goals of patient work, that is, due to shortages of time or staff and leading to stress of conscience.^16^

In this study, we investigate healthcare personnel’s long-term experience of work intensification and its relation to stress of conscience. Specifically, long-term work intensification in this study refers to employees’ subjective perceptions of several years of work intensification in their workplace.^4,6,15^ In healthcare, work intensification has been connected to care not being provided, missed care and compromised patient safety, as personnel need to rationalize care and therefore cannot provide all the care patients require.^17,18^ In addition, in the long run, work intensification may exceed personnel’s resources, causing stress^19^ and fatigue, which, in the healthcare context, can further impair quality of care.^20^ Therefore, we propose that long-term work intensification may lead more often to personnel’s appraisal of falling short of the ethical standards of their tasks evoking more stress of conscience.^11^ Therefore, we tested the following hypothesis:

Hypothesis 1: High long-term work intensification is associated with high stress of conscience among healthcare workers.

### Managing organizational change in intensified working life

In the present study, the organization under investigation was undergoing a major change and change management was under scrutiny. In this study, change management refers to the handling and communicating of the change process, not to the inauguration of the new hospital building, patient-centred service and care processes. Thus, the concept of change management relates to the personnel’s evaluation of how management and supervisors solved problems emerging during the organizational change, and whether management provided adequate support for developing personnel’s competence. Effective change management is an important tool in implementing successful organizational change.^21^ However, many hospital improvements encounter challenges or even fail.^22^ In the present study, change management was examined longitudinally with regard to challenges from long-term work intensification during the COVID pandemic. This yields new knowledge of change management concerning stress of conscience in healthcare during a challenging phenomenon.

It is reasonable to assume that the association between long-term work intensification and healthcare personnel’s stress of conscience is mediated through change management during organizational change. Work intensification can render successful change management more difficult because prolonged work intensification may already have exceeded management’s resources.^6,23^ During organizational change*,* managers often have more tasks than usual in concurrently managing the organization and the major change.^24,25^ Accumulating demands may, according to conservation of resources theory,^26^ lead to a resource loss cycle among managers impairing their capacity and energy to support employees,^27^ solve problems and ensure employees’ competence during the change. Work intensification may consequently have an adverse effect on personnel’s experience of change management*.*

Poor change management, in turn, may be associated with personnel’s greater stress of conscience. Little or no support during organizational change can be a resource loss according to conservation of resources theory.^26^ Therefore, if personnel lack adequate resources to provide a proper quality of patient care,^2^ this may lead to stress of conscience.^11^ More specifically, if problems emerging during the organizational change were inadequately resolved and if personnel’s competence is not appropriately addressed, the resulting adverse effect on patient care may serve to exacerbate stress of conscience.^11^ In addition, low perceived organizational support has been related to high moral distress among nurses.^28^ This reasoning led us to test the second hypothesis of the study:

Hypothesis 2: The association between high long-term work intensification and high stress of conscience is mediated through poor change management.

### COVID-19 as an additional risk for stress of conscience during work intensification

The COVID-19 pandemic influenced healthcare systems causing healthcare professionals major psychological stress.^10,12,29^ In this study, COVID-19-related work stress was defined as stress symptoms which employees themselves reported to be caused by the COVID-19 pandemic. COVID-19-related work stress has been attributed to many causes, including fear of becoming infected and transmitting the virus to significant others^30^ and increase in workload.^2^

Even before COVID-19, the health sector was characterized by high work intensification depleting employees’ resources.^2,3,16^ During the pandemic, work-related stress increased healthcare workers’ errors at work due to cognitive failures.^31^ Cognitive failures cause more patient safety incidents^32^ and adverse events among patients.^33^ Failures and adverse events conflicted with employees’ moral intention to provide good care^34^ triggering stress of conscience.^11^ Consequently, it is reasonable to suppose that COVID-19-related work stress strengthened the relationship between long-term work intensification and stress of conscience, meaning that the relationship between work intensification and stress of conscience would be stronger if an employee experiences higher COVID-19-related work stress. Hence, we tested the following hypothesis:

Hypothesis 3: COVID-19-related work stress moderates (strengthens) the association between long-term work intensification and stress of conscience.

### In summary

In healthcare, long-term work intensification,^6^ organizational changes^35^ and crises like the COVID-19 pandemic^30^ are major challenges that need to be studied in relation to stress of conscience to ascertain how risky they are for personnel well-being. This is because stress of conscience can be used to determine if personnel can or cannot, according to their moral standards, adequately perform their duties in patient care.^1,11^ Therefore, in relation to the major challenges studied, stress of conscience can be seen as a noteworthy and new indicator for organizational improvements and interventions to address the situations leading to ethical failures in patient care.

## Method

### Participants and procedure

Participants were healthcare personnel doing patient care work in a large healthcare district in Finland, which consisted of a central hospital and several public health centres. The organization underwent a major organizational change between 2019 and 2021, which included relocating the hospital services to a newly built hospital building and a transition from a traditional organizational model to a patient-centred service model. The first electronic questionnaire was sent via the organization’s representative to all employees in September 2019 (T1), prior to the COVID-19 pandemic, when the organizational change was at an early phase. The emails included a description of the project, information about the voluntary nature of participation, assurances of confidentiality, collection and use of personal data and the link to the electronic survey. The respondents gave their informed consent to participate by responding to an accompanying item before they were able to continue further in the survey. The participants’ responses were returned directly to the university researchers. In 2019, there were 1,024 participants (response rate 25%).

The second questionnaire was sent in September 2021 (T2), while the pandemic was ongoing and the organizational change was completed, meaning that most of the personnel had moved to the new hospital unit and the new organizational model had been implemented. The survey was sent only to those participants (*n* = 571) who in 2019 had given permission and a personal email address for further inquiry. Otherwise, the data collection procedure was similar to T1. In 2021, there were 318 participants (response rate 56%), including several occupational groups. In the present study, we investigated only those participants who were in direct patient work, therefore 95 respondents were excluded. A further 12 of the remaining respondents were no longer working in the healthcare district. Therefore, 211 participants were left as the final sample.

### The final sample of the study

The sample of the present study included 211 healthcare employees who had responded to the survey both in 2019 and in 2021, and who were working in the same healthcare district at the second measurement point. At T1, 89% of the participants were women, and the largest age group was those aged 41-45 years. In the sample, 58% of participants were doing day work and 42% shift work. The participants consisted of nurses (*n* = 127, 60%), physicians (*n* = 19, 9%), mental health workers (*n* = 13, 6%), rehabilitation workers (*n* = 11, 5%) and others, such as social workers, specialists etc. (*n* = 41, 19%).

### Dropout analysis

We performed a dropout analysis, which showed that between the final sample (*n* = 211) and those who participated only at baseline (*n* = 813), there was no significant difference in gender [ χ^2^(1) = 0.71, *p* = .40] or age [ χ^2^ (8) = 7.65, *p* = .47]. However, there were more shift workers in the final sample than in the baseline study [ χ^2^(1) = 28.74, *p* < .00]. The final sample did not differ as regards stress of conscience from those who participated only at baseline [*t*(1004) = -1.13, *p* = .26, Cohen’s d = -0.09] or in work intensification [*t*(1018) = -0.59, *p* = .55, Cohen’s d = -0.05]. Thus, the participants in the final sample represented the baseline respondents relatively well.

### Ethical considerations

The study was implemented throughout according to the guidelines of the Finnish National Board on Research Integrity and the 1964 Helsinki Declaration and its later amendments or comparable ethical standards. The Finnish instructions were that no ethics committee review was necessary because participation was voluntary, informed consent was requested and participants were assured that they were free to withdraw from the study at any time. In the study, no personal health data were collected nor was precise age elicited. Participants were guaranteed confidentiality and assured that the data would be used in compliance with the European Union General Data Protection Regulation.

### Measures

*Stress of conscience* was measured at T1 and T2 with the Stress of Conscience Questionnaire (SCQ).^11^ We used a shortened version of the SCQ, which has been recently validated in the healthcare context and items of the shortened SCQ scale are available in that study.^35^ The SCQ first measures frequency of situations troubling employee’s consciences (6 items) and elicits the magnitude of stress caused by these situations (6 items). For example, the first question (frequency) was ‘How often do you lack the time to provide the care the patient needs?’ on a scale from 1 (never) to 6 (daily), and the second question (magnitude) was ‘Does this give you a troubled conscience?’ on a scale 1 (not at all) to 6 (very much). Third, frequency and magnitude were multiplied resulting to six items describing the respondent’s stress of conscience. The scale of the score varied from 1 to 36, higher scores indicating higher levels of stress of conscience. Fourth, the mean score from the multiplied items was calculated for further analysis. In the analysis, stress of conscience at T2 was the dependent variable and stress of conscience T1 was the control variable.

*Long-term work intensification* was measured with the 5-item Intensification of Job Demands Scale.^10^ The Intensification of Job Demands Scale measures work intensification, which refers to the increasing amount of effort an employee needs to invest during the working day, consisting of ‘need to work at increasing speed, perform tasks simultaneously or reduce idle time’.^5(p899)^ At T1, the instructions for the work intensification questions began with ‘In the last 5 years…’ and continued with the statements, for example, ‘…even more work has to be accomplished by fewer and fewer employees’ and ‘…it is increasingly harder to take time for breaks’. The same five questions were repeated at T2. The only modification was the time interval in the response instruction, which was changed from 5 years to 2 years (‘In the last 2 years…’). This modification was made because the follow-up period of the study was 2 years. The response scale varied from 1 (not true at all) to 5 (completely true) at both time points. Because we were trying to capture the long-term experience of work intensification, we calculated a total long-term work intensification score based on the sum of T1 and T2 mean scores (scale 2–10).

*Change management* was measured at T2 with four items from the Meaningful Organizational Change Scale.^36^ The scale measures how personnel experience management actions during organizational change. The questions were, for example, ‘The organization’s management has actively solved problems that occurred during the organizational change’ and ‘My superior has ensured enough support for developing my competence after the change’. In all statements, response scales were from 1 (very poorly) to 5 (very well). A mean score sum variable was calculated from the items on a scale 1–5.

*COVID-19-related work stress* was measured at T2 by first defining psychological stress as a situation in which a person feels anxious, alarmed, nervous or uneasy, or may have difficulties sleeping because of issues constantly troubling them, and then asking to what extent the COVID-19 pandemic had caused participants such stress at work. The question was formulated on the basis of the single-item psychological stress measure developed by Elo et al.^37^ and adapted to the pandemic context. The response scale ranged from 1 (not at all) to 5 (very much).

*Controlled background variables* in the statistical analyses were gender (1 = female and 2 = male), age T1 (nine categories) and work shift T2 (1 = daytime work and 2 = shift work). The control variables were chosen because older age and male gender have been found to be protective against stress of conscience and because shift work has been found to be connected to increased stress of conscience.^16^

A summary of measures, means, standard deviations and Cronbach’s alphas of variables are presented in Table 1.

**Table 1.** Summary table of measures and descriptive statistics (M, SD) among healthcare personnel (*n* = 211) concerning seven study variables.

Scale	Cronbach’s alphas	M (SD)	No. of items	Scale-range
Stress of conscience T1	.89	12.87 (6.64)	6 x 6	1–36^a^
Stress of conscience T2	.88	12.60 (7.01)	6 x 6	1–36^a^
Work intensification T1	.91	3.74 (1.06)	5	1–5^a^
Work intensification T2	.92	3.33 (1.21)	5	1–5^a^
Long-term work intensification T1 + T2	.91	7.07 (1.94)	10	2–10^a^
Change management	.85	2.75 (.96)	4	1–5^b^
COVID-19-related work stress T2	-	2.74 (1.05)	1	1–5^a^

^a^Higher score indicates more of the given construct.

^b^Higher score indicates better change management.

### Statistical analysis

For the statistical analyses, we used Statistical Package for Social Sciences (SPSS) (version 27) and Hayes’ PROCESS macro for SPSS (version 4.0). First, we used Pearson’s correlations to investigate the relations between variables. Second, we used the PROCESS macro to conduct moderated mediation analysis. Third, we generated simple slope diagrams for the significant moderation results.

In our study, we used Haynes’^38^ moderated mediation Model 5 (see Figure 1), which describes the direct effect between X and Y, mediation from X through M to Y and the moderator effect of Z between X and Y. Model 5 also shows the direct effect between Z and Y and the control variables. The controlled background variables were gender, age and day/shift work. We controlled for stress of conscience at T1 to consider its effect on Y (stress of conscience at T2). In addition, the main effect of Z (COVID-19-related work stress) on Y (stress of conscience T2) was controlled.

Table 2 presents the Pearson’s correlations between variables. Long-term work intensification (T1 + T2) correlated positively with stress of conscience at T2 and negatively with change management at T2. Poor change management at T2 correlated positively with stress of conscience at T2. COVID-19 stress at T2 correlated positively with stress of conscience at T2. Stress of conscience showed relatively high stability over a two-year follow-up time and therefore stress of conscience at T1 was controlled for stress of conscience at T2 (dependent variable) in our moderated mediation model.

**Table 2.** Pearson’s correlations of the study variables among healthcare personnel (*n* = 211).

Variables	1	2	3	4	5	6	7	8	9	10
1. Stress of conscience T1	-									
2. Stress of conscience T2	.57***	-								
3. Gender (f/m)	-.12	-.12	-							
4. Age	-.12	-.14*	-.04	-						
5. Shift work (d/s)	.15**	.24***	.01	-.09	-					
6. Work intensification T1	.49***	.35***	-.01	.05	.15*	-				
7. Work intensification T2	.35***	.53***	-.11	.09	.36***	.46***	-			
8. Long-term work intensification (T1 + T2)	.48***	.53***	.05	.08	.31***	.83***	.87***	-		
9. Change management T2	-.23***	-.37***	-.05	.00	-.11	-.21**	-.34***	-.33***	-	
10. COVID-19 stress T2	.28***	.36***	-.04	-.02	-.00	.15*	.19**	.20**	-.13**	-

^*^p < .05. **p < .01. ***p < .001. Gender (f/m) = female/male. Age was measured with nine age groups 1 = 25 years or under, 2 = 26–30, 3 = 31–35, 4 = 36–40, 5 = 41–45, 6 = 46–50, 7 = 51–55, 8 = 56–60 and 9 = over 60 years. Work shift (d/s) = day/shift work.

## Results

### Direct effects

**Figure 2.**
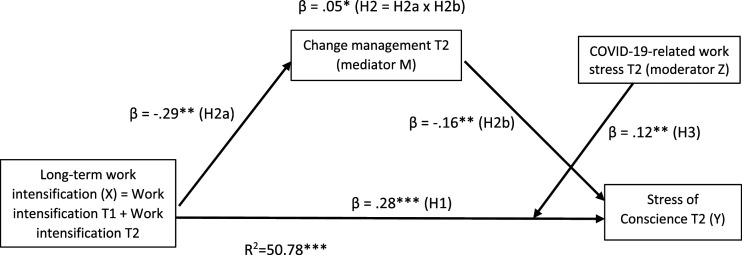
Moderated mediation analysis results of the relationships between long-term work intensification, change management, COVID-19 stress and stress of conscience among healthcare personnel (*n* = 211). * Significant in 95% confidence interval [0.01, 0.09], ** *p* < .01, *** p < .001.

The moderated mediation analysis between study variables showed that the hypothesized model explained 50.78% (*p* < .001) of variance in stress of conscience at T2. The analysis showed, in line with our hypothesis (H1), that there was a significant positive direct effect between long-term work intensification and stress of conscience [β = 0.28, *t*(202) = 5.28, *p* < .001] (see Figure 2).

The control variables showed some significant direct effects on stress of conscience at T2. Stress of conscience at T1 significantly predicted stress of conscience at T2, indicating relatively high stability for the construct (β = 0.30, *t*(202) = 4.06, *p* < .001). COVID-19-related work stress was related to stress of conscience at T2 (β = 0.18, *t*(202) = 3.02, *p* < .01). Older age was associated with lower stress of conscience [β = -0.13, *t*(202) = -2.29, *p* < .05]. Gender and shift work showed no significant associations.

### Mediation effect

Indirect effect of long-term work intensification through change management at T2 on stress of conscience at T2 was significant (β = 0.05, SE = 0.02) with a 95% confidence interval [0.01, 0.09], thereby supporting H2 (see Figure 2). Thus, change management had a partial mediation effect between work intensification and stress of conscience: there was an independent process in which work intensification showed a significant association with poor change management [β = -0.29, *t*(205) = -3.32, *p* < .01]. Poor change management (i.e. low scores), in turn, showed a significant negative association with high stress of conscience [β = -0.16, *t*(202) = -2.96, *p* < .00].

### Moderation effect

**Figure 3.**
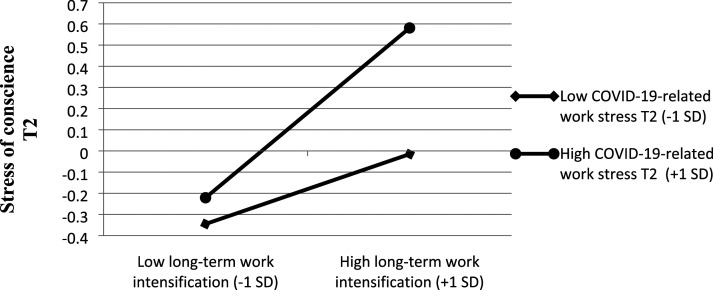
Interaction effect of ‘long-term work intensification*COVID-19-related work stress’ on stress of conscience among healthcare personnel.

COVID-19-related work stress at T2 significantly moderated the direct effect of long-term work intensification on stress of conscience at T2 (β = 0.12, *p* < .01, ΔR^2^ = 0.02, *p* < .01), after controlling for the main effect of stress of conscience at T1 [β = 0.18, *t*(202) = 3.02, *p* < .00] (Figure 2). According to the simple slope analysis, stress of conscience was highest when work intensification and COVID-19 work stress were both high (+1 *SD*), which supports H3 (see Figure 3). Among the participants with high COVID-19 work stress (*+1 SD*), the association between long-term work intensification and stress of conscience level was stronger (β = 0.40 *p* < .01) than among those with low COVID-19 work stress (*-*1 *SD,* β = 0.16, *p* < .05). Thus, COVID-19-related work stress strengthened the negative relationship between work intensification and stress of conscience.

## Discussion

The aim of this two-year longitudinal study was to investigate the factors contributing to stress of conscience among the personnel of a large healthcare organization. The study was conducted during a period of major organizational change and the outbreak of the COVID-19 pandemic. The following three hypotheses were tested: Hypothesis 1, higher long-term work intensification is associated with higher stress of conscience, Hypothesis 2, the association of long-term work intensification with stress of conscience is mediated through poor change management and Hypothesis 3, COVID-19-related work stress strengthens the association between long-term work intensification and stress of conscience.

The first hypothesis (H1) was supported: high long-term work intensification was related to high stress of conscience. Long-term work intensification may be interpreted as a longstanding and accumulating job hindrance^6^ exceeding employees’ resources to carry out patient work tasks properly.^17,18^ In this study, long-term intensification referred to increased time pressures on employees, pace of work and multitasking demands over many years.^5,6^ The result is alarming because there are no signs that work intensification^6^ and its impact on stress of conscience in the healthcare context will decrease in the near future.^39^ Prolonged work intensification may morally exhaust healthcare personnel with undone tasks, care not provided and patient safety compromised^2^ and adversely affect personnel's well-being because stress of conscience is connected to more severe burnout.^39^

Another main finding of the study was that the association of high long-term work intensification with high stress of conscience was partially mediated through poor change management, which supported Hypothesis 2. This result may indicate that management’s handling and communicating of the change process may have weakened because work intensification exceeds management's and supervisors' capacity to manage all aspects of organizational change properly.^23,24,27^ On the other hand, the capacity of personnel to absorb information related to change may also be limited in intensifying care work.^35^ These are the risks of successful change management in an ever-intensifying healthcare sector. Therefore, ensuring adequate resources for management, supervisors and staff is crucial for successful change management.

Finally, COVID-19-related work stress was found to strengthen the association between long-term work intensification and stress of conscience, thereby verifying Hypothesis 3. The finding may indicate that personnel encountering high prolonged work intensification literally reached the limit of their resources to provide good care while experiencing sudden high COVID-19-related work stress. The result shows that because of work intensification healthcare workers are vulnerable when confronting unforeseen crises like the COVID-19 pandemic. The conclusion concurs with the findings of Willis et al.^2^ that COVID-19 merely served to expose the flaws already present in the healthcare system. In some countries, such as India and Brazil, the COVID-19 pandemic drove the healthcare systems to the verge of collapse,^40,41^ which bodes ill for workers’ stress of conscience. Therefore, long-term work intensification should be tackled by any means and resources available to protect the quality of patient work and alleviate personnel’s stress of conscience.

In conclusion, because higher long-term work intensification was related to higher stress of conscience, and because this relationship was exacerbated by higher COVID-related work stress, long-term work intensification in healthcare may be one of the main factors predisposing to poorer quality in patient care, compromised patient safety and adverse events among patients.^17,18^ Furthermore, work intensification can be an additional risk factor increasing personnel’s vulnerability to other sudden risks such as pandemics,^10^ all triggering stress of conscience.^11^ From this standpoint, stress of conscience could be seen as an indicator for improvements in healthcare organizations to maintain ethical standards of patient care that the employees themselves can subscribe to.^42,43^

### Limitations

There are a few noteworthy limitations in this study. The response rate of the baseline survey was relatively low (25%), even though the follow-up survey resulted in a better response rate (56%). This may limit the generalizability of the results and also relate to a major organizational change occurring during the follow-up, eroding staff’s response motivation. Dropout analysis showed that there were more shift workers in the final sample than in the baseline study, which may have some impact on the results because shift work has been found to be related to more severe stress of conscience.^3^ Additionally, the research was based on self-report questionnaires and although it was a longitudinal study, no causal inferences can be made. Finally, we are not sure how well the results can be generalized to countries other than Finland, although work intensification in healthcare seems to be common globally.^17,18^

### Recommendations for future research

Research on how to reduce long-term work intensification is urgently needed; it is a risk for high stress of conscience and poor change management in healthcare organizations. A study on the antecedents of work intensification has shown that job autonomy and supervisory support are associated with lower work intensification,^44^ and it would be wise to increase these job resources, also in healthcare, to tackle work intensification. Moreover, research to improve and support change management under the effects of work intensification is needed to make sure that organizational changes in healthcare are implemented in the most effective way even though management’s resources are meagre. Finally, investigation of how to prepare for an unexpected crisis like a pandemic in healthcare is needed to strengthen the healthcare system’s resources to alleviate stress of conscience.

### Implications

#### Using stress of conscience as an indicator for organizational improvements

Healthcare workers’ stress of conscience should be seen as an indication for make system improvements and interventions to the external sources of stress of conscience, not just to improve the resilience of workers. The results of our study suggest that one such implication would be a major increase of resources for healthcare workers and management to cope with and tackle long-term work intensification and organizational changes, which are significant risk factors related to high stress of conscience.

#### Organizational change needs adequate resources

Under the influence of long-term work intensification successful change in healthcare requires managers to be provided with additional time, resources and support. Therefore, if adequate resources are allocated for management, workers may perceive change management more successful and supportive, which may help to alleviate their stress of conscience.

#### Emergency resources for crisis

Because work intensification has brought healthcare workers to the limit of their resources, in preparation for possible new pandemics, healthcare organizations need emergency plans and resources that could be deployed in time of crisis to alleviate stress of conscience. The WHO has a toolkit for assessing a healthcare system’s capacity for crisis management,^8^ which could be used as a guideline.
